# Evaluating scientific research barriers by gender and other characteristics from the perspective of ophthalmologists in Turkey: A multicenter survey study

**DOI:** 10.1371/journal.pone.0273181

**Published:** 2023-01-25

**Authors:** Burak Erdem, Abdulkadir Obut, Mehmet Kay, Mustafa Gok, Sedat Bostan

**Affiliations:** 1 Department of Ophthalmology, Ordu University Faculty of Medicine, Ordu, Turkey; 2 Department of Ophthalmology, Afyon State Hospital, Afyon, Turkey; 3 Department of Ophthalmology, Sancaktepe Sehit Prof Dr Ilhan Varank Training and Research Hospital, Istanbul, Turkey; 4 Department of Ophthalmology, Private Atanur Eye Hospital, Isparta, Turkey; 5 Department of Health Management, Ordu University Faculty of Health Sciences, Ordu, Turkey; Public Library of Science, UNITED KINGDOM

## Abstract

**Background/Aim:**

The ever-increasing population and life expectancy worldwide increase the prevalence of ophthalmic diseases, and the need for ophthalmic research expands accordingly. In our study, we aimed to evaluate many aspects of the barriers, especially gender disparities, confronting ophthalmologists who aspire to conduct scientific research (SR).

**Materials and methods:**

In this descriptive quantitative study, we distributed an online questionnaire to ophthalmologists in Turkey with 21 questions presented on a five-point Likert scale and two open-ended questions. The survey was prepared with Google forms. Participants were recruited via e-mail and social networks. A multicenter survey was conducted between January 29 and February 20, 2021, and a total of 210 valid responses were recorded.

**Results:**

Participants’ responses were grouped into four types of barriers: motivation, time constraints, research support, and competence. Participants’ motivation to conduct research was above average (3.54±0.96), but most stated that they have time constraints (3.74±0.97). Participants did not agree that there is adequate support for research (2.35±0.76), and they self assessed their level of the required competence to be average (2.87±1.08). Women were more motivated to do SR than men (p = 0.008), but there were no statistically significant differences between women and men in terms of time constraints, research support, and level of competence (p = 0.853, p = 0.482, and p = 0.558, respectively). Although there is no statistically significant difference between men and women regarding time constraints, female physicians mentioned more about the barriers arising from their personal responsibilities (p = 0.038).

**Conclusions:**

Our study revealed that ophthalmologists are enthusiastic about doing SR but encounter obstacles with regard to time availability and research support. In addition, there is a need to reinforce competence in SR. Although female physicians are more motivated than men, they must deal with competing domestic responsibilities.

## 1. Introduction

For physicians, participating in scientific research (SR) represents a commitment to contributing to science that involves tremendous effort and meticulous time management. Creating and publishing research requires knowledge, experience, and institutional support—financial incentives, technical facilities, and trained personnel [1]. On the other hand, as Kothari points out, research is the art of scientific investigation [2].

The importance of research productivity in ophthalmology will increase in the future. Age-related eye diseases are the main causes of visual impairment worldwide, and the prevalence of ophthalmic diseases is directly proportional to the constantly aging population and higher life expectancy [3]. This puts an additional burden on the healthcare system and its associated costs [4], and people’s quality of life diminishes due to eye diseases [5]. Therefore, academic activities in the field of ophthalmology should be progressing accordingly to address these conditions.

In recent years, publication productivity has increased steadily in Turkey in line with the global trend. At present, an analysis of Web of Science (WoS) data found 781,262 articles addressing Turkey, of which approximately 38% were published between 2015 and 2020. Likewise, we found that about 39% of the total of 8,622 articles published from Turkey in the last five years related to ophthalmology categories; Turkey ranks twelfth among all countries. Although these data do not provide detailed information, they reflect the existing situation of publication productivity in the field of ophthalmology in Turkey.

Research on the position, status, and influence of women academics has attracted more and more attention from researchers in recent years [6]. Accordingly, the number and impact of women in the academic community are gradually increasing. Nevertheless, gender disparities in academic leadership positions persist [7]. In particular, the gender gap is felt much more in surgical areas [8]. In the field of ophthalmology, gender disparities also manifest themselves in academic leadership positions [9]. Consequently, women have not yet caught up with men in the number of scientific publications [10].

Researchers often conduct bibliometric studies to reveal the current state of scientific performance [11], but bibliometric studies are insufficient for compiling the information required to increase research productivity. Development in research is a continuum, meaning that the efforts to increase research productivity must be constantly up-dated. For this reason, survey studies to pinpoint the obstacles deterring researchers, the lead actors of SR, are the obvious approach [12–14], but few studies using this method in ophthalmology have been published. We found no such study in the literature for ophthalmologists in Turkey.

The aim of this study was to evaluate the barriers and the incentives for SR from the perspective of ophthalmologists in Turkey.

## 2. Materials and methods

### 2.1. Research sample and data collection

In this descriptive quantitative study, ophthalmologists—residents, specialists, and academicians—were recruited with an online survey to identify barriers to SR. The study was conducted with the approval of the Ordu University Ethics Committee. The survey was prepared with Google forms. The link to access the survey was sent to ophthalmologists via e-mail or on social networks. The participants’ e-mail addresses were gathered from the Turkish Ophthalmology Association’s public website accessible to all. Although 6460 ophthalmologists are registered here, we distributed our questionnaire to 1500 ophthalmologists whose contact information is up-to-date and who reside in Turkey. During the data collection period between January 29 and February 20, 2021, a total of 210 valid questionnaires were recorded.

The current survey consisted of two parts: demographic characteristics and a scale of ophthalmologists’ perception of research barriers.

#### 2.1.1. Demographic characteristics

This section includes personal and institutional characteristics of the respondents. We enquired about the participants’ age, gender, employment position, marital status, year of practice as an ophthalmologist, institution type, geographic region, and training.

#### 2.1.2. Research barriers scale

This scale was prepared after a detailed literature review and sent to five ophthalmologists for review and feedback. It consists of 21 items with Likert-type responses and two open-ended questions. We intended to examine the extent to which ophthalmologists are constrained in their SR by several impediments with this scale.

The Turkish version of the survey is available in S1 Table.

The English version of the survey is available in S2 Table.

### 2.2. Statistical analysis

IBM SPSS for Windows (version 20.0; IBM-SPSS, Chicago, IL, USA) software was used for statistical analysis. The Kaiser-Meyer-Olkin (KMO) test was used to determine adequate sample size. In addition, according to the Bartlett’s test results, the correlation between items was significant at the 0.001 level. The “principal components” method was used in factor analysis to understand the construct validity of the items of the scale. “Varimax” rotation was used to transform the components into more clearly interpretable factors. In the scale of 21 questions, three were removed due to inconsistency in factor analysis. The scale was validated as 18 expressions. It was observed that the scale items had factor loads between 0.414 and 0.852. The scale of 18 statements was categorized into four factors—motivation (4 statements), research support (7 statements), time constraints (3 statements) and competence (4 statements). For the reliability analysis of the study, Cronbach’s alpha coefficient was applied, and it was found that this value was reliable with 0.771. A t-test and ANOVA were used to evaluate the participants’ responses according to demographic characteristics. Tukey’s test was used to evaluate the differences between groups in post hoc analysis.

## 3. Results

The demographic characteristics of 210 ophthalmologists participating in the study are presented in Table 1. Women accounted for 43.3% of participants and 56.7% were men. In total, 15.7% were residents, 60.0% were specialists, and 24.3% were academicians. Seven geographical regions were examined in four sections according to the level of socio-economic development. The Marmara region is specified as the first zone, the Central Anatolia region as the second zone, the Aegean and Mediterranean regions as the third zone, and the Black Sea, Eastern Anatolia, and Southeastern Anatolia regions make up the fourth zone. The first zone has the highest level and the fourth zone has the lowest level of socio-economic development.

**Table 1:** Demographic characteristics of the participants.

VARIABLE	N	%
**1. Gender**		
Female	91	43.3
Male	119	56.7
**2. Age**		
25–34	100	47.6
35–44	80	38.1
45 and over	30	14.3
**3. Title**		
Ophthalmology Residency	33	15.7
Ophthalmology Specialist	126	60.0
Academicians (Assistant Professor & Associate Professor & Professor)	51	24.3
**4. Marital status**		
Married	155	73.8
Single	55	26.2
**5. Working year as an ophthalmologist**		
1–5 years	47	22.4
6–10 years	66	31.4
11–15 years	51	24.3
16 and up	46	21.9
**6. Institution**		
State Hospital	124	59.0
University Hospital	42	20.0
Private Hospital	44	21.0
**7. The geographic region of your institution**		
First Zone (Marmara Region)	71	33.8
Second Zone (Central Anatolia Region)	42	20.0
Third Zone (Aegean Region & Mediterranean Region)	50	23.8
Fourth Zone (Black Sea Region & Eastern Anatolia Region & Southeastern Anatolia Region)	47	22.4
**8. The status of the institution where you completed the residency training**		
State Hospital	90	42.8
University Hospital	120	57.1
**9. The geographical region of the institution where you completed the residency training**		
First Zone (Marmara Region)	87	41.4
Second Zone (Central Anatolia Region)	60	28.6
Third Zone (Aegean Region & Mediterranean Region)	42	20.0
Fourth Zone (Black Sea Region & Eastern Anatolia Region & Southeastern Anatolia Region)	21	10.0

**Fig 1:**
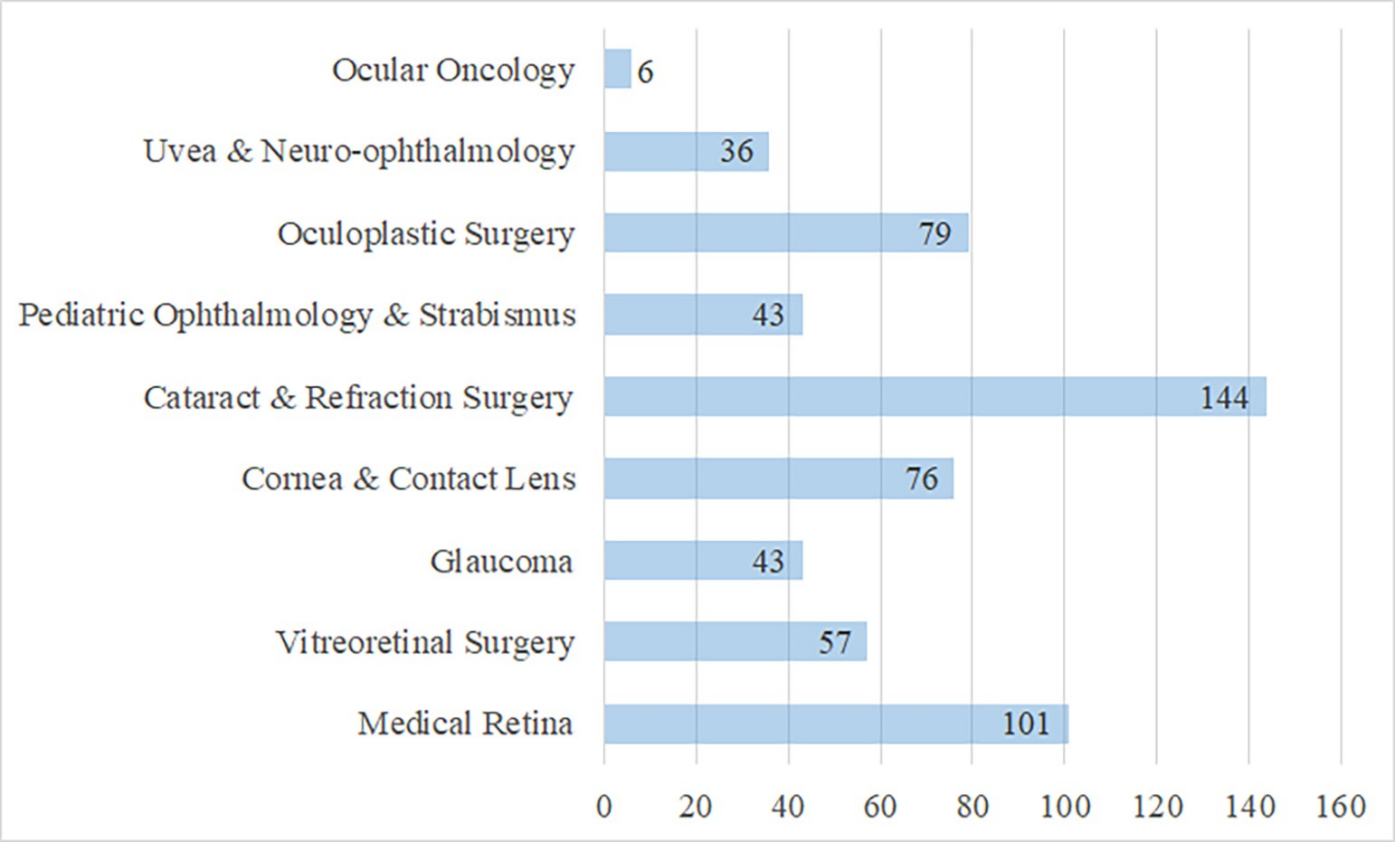
Distribution of the participants according to their ophthalmological interests.

The distribution of participants according to their ophthalmological interests is presented in Fig 1. In this section, participants could choose more than one option. It was observed that most participants are interested in cataract and refraction surgery and medical retina. Interest in neuroophthalmology and ocular oncology was minimal compared to other fields.

**Fig 2:**
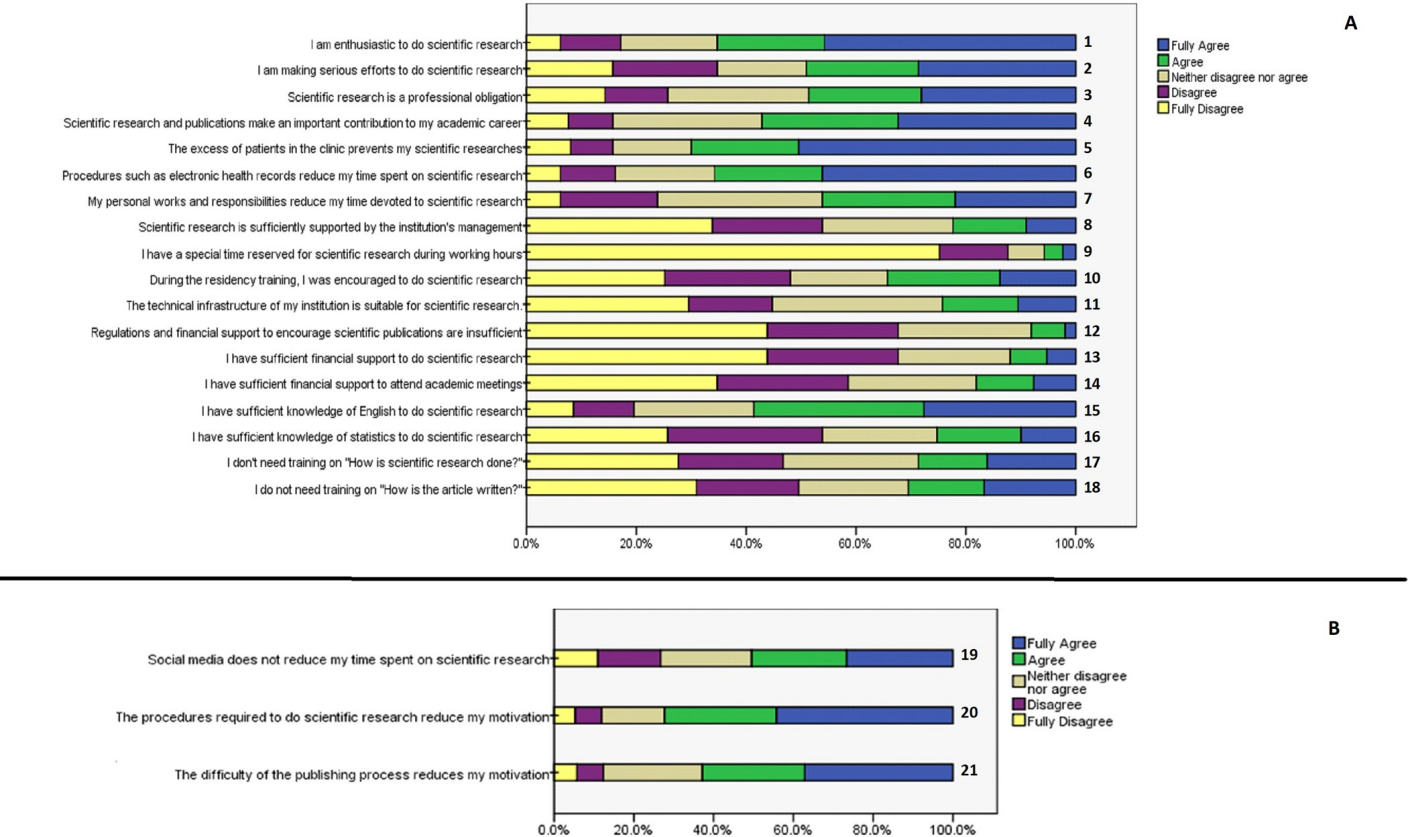
Likert bar graphs showing the distribution of the answers given by the participants to the questions. (A) Questions inside the scale after factor analysis. (B) Questions outside the scale.

To understand the obstacles faced by ophthalmologists who wish to perform SR, the frequency distribution of the research barriers, as reported on the five-point Likert scale and evaluated in the four factors listed above, is presented in Table 2. Likert bar graphs are given in Fig 2 to visualize the distribution of responses to questions within and outside the scale after factor analysis.

**Table 2:** Frequency distribution of the research barriers scale of ophthalmologists.

Expression	Fully disagree		Disagree		Neither disagree nor agree		Agree		Fully agree		Mean	SD
	N	%	N	%	N	%	N	%	N	%		
**Motivation**											**3.54**	**0.96**
I am enthusiastic to do scientific research	13	6,2%	23	11,0%	37	17,6%	41	19,5%	96	45,7%	3.87	1.27
I make serious efforts to do scientific research	33	15,7%	40	19,0%	34	16,2%	43	20,5%	60	28,6%	3.27	1.45
Scientific research is a professional obligation	30	14,3%	24	11,4%	54	25,7%	43	20,5%	59	28,1%	3.36	1.37
Scientific research and publications make an important contribution to my academic career	16	7,6%	17	8,1%	57	27,1%	52	24,8%	68	32,4%	3.66	1.22
**Time Constraints**											**3.74**	**0.97**
The excess of patients in the clinic prevents my scientific researches	17	8,1%	16	7,6%	30	14,3%	41	19,5%	106	50,5%	3.96	1.29
Procedures such as electronic health records reduce my time spent on scientific research	13	6,2%	21	10,0%	38	18,1%	41	19,5%	97	46,2%	3.89	1.26
My personal works and responsibilities reduce my time devoted to scientific research	13	6,2%	37	17,6%	63	30,0%	51	24,3%	46	21,9%	3.38	1.18
**Research Support**											**2.35**	**0.76**
Scientific research is sufficiently supported by the institution’s management	71	33,8%	42	20,0%	50	23,8%	28	13,3%	19	9,0%	2.43	1.31
I have a special time reserved for scientific research during working hours	158	75,2%	26	12,4%	14	6,7%	7	3,3%	5	2,4%	1.45	0.93
During the residency training, I was encouraged to do scientific research	53	25,2%	48	22,9%	37	17,6%	43	20,5%	29	13,8%	2.74	1.39
The technical infrastructure of my institution is suitable for scientific research	62	29,5%	32	15,2%	65	31,0%	29	13,8%	22	10,5%	2.60	1.32
Regulations and financial support to encourage scientific publications are sufficient	92	43,8%	50	23,8%	51	24,3%	13	6,2%	4	1,9%	1.98	1.00
I have sufficient financial support to do scientific research	92	43,8%	50	23,8%	43	20,5%	14	6,7%	11	5,2%	2.05	1.17
I have sufficient financial support to attend academic meetings	73	34,8%	50	23,8%	49	23,3%	22	10,5%	16	7,6%	2.32	1.26
**Competence**											**2.87**	**1.08**
I have sufficient knowledge of English to do scientific research	18	8,6%	23	11,0%	46	21,9%	65	31,0%	58	27,6%	3.58	1.23
I have sufficient knowledge of statistics to do scientific research	54	25,7%	59	28,1%	44	21,0%	32	15,2%	21	10,0%	2.55	1.29
I don’t need training on "How is scientific research done?"	58	27,6%	40	19,0%	52	24,8%	26	12,4%	34	16,2%	2.70	1.41
I do not need training on "How is the article written?"	65	31,0%	39	18,6%	42	20,0%	29	13,8%	35	16,7%	2.66	1.45

SD; Standart deviation

The open-ended questions asked participants to list the three most motivating and most discouraging aspects of SR. Respondents stated that the strongest motivators were desire to contribute to science and make their mark, curiosity and desire to learn, and earning the prestige of academic titles. The most discouraging factors reported were clinical workload and excess of patients, insufficient technical infrastructure, and the general difficulties of research and publishing procedures, such as official approvals, from the beginning through to the final stages of a project.

When we looked at research barriers by gender, we found no difference between men and women in terms of time constraints, research support, and competence (p = 0.853, p = 0.482, and p = 0.558, respectively). However, women are definitely more motivated than men to do SR (p = 0.008). In Table 3, the answers given on research barriers scale by gender are shown under four main headings. When we examined the questions one by one, we observed a statistically significant difference between men and women for questions 1, 4, 7, 16, and 21 (p = 0.035, p = 0.001, p = 0.038, p = 0.034, and p = 0.020, respectively)(see Fig 2 for numbered questions). Accordingly, women are more enthusiastic than men to do SR and think that scientific studies contribute more to their academic careers. However, women stated that they had more personal responsibilities. Also, they stated that the challenges of the publishing process deterred their motivation, and they had less statistical knowledge than men.

**Table 3:** Answers are given to the research barriers scale by gender.

	Female		Male		
	Mean	SD	Mean	SD	P
**Motivation**	3.74	0.93	3.39	0.97	**0.008**
**Time Constraints**	3.76	0.92	3.74	1.01	0.853
**Research Support**	2.32	0.75	2.39	0.78	0.482
**Competence**	2.83	1.06	2.92	1.11	0.558

SD; Standart deviation

Considering the age factor, physicians aged 45 and over stated that they are less motivated than younger physicians (p = 0.008). Those between the ages of 25 and 34 stated that they had less competence than other physicians, as expected (p = 0.023). There was no difference between age groups in terms of research support (p = 0.392) and time constraints (p = 0.601).

According to academic title, residents and academicians seem more motivated than specialist doctors (p = 0.001). Although the academicians stated that they experienced less problem in terms of time constraints compared to the other two groups, this difference was not statistically significant (p = 0.084). Specialists stated that they had more difficulty in obtaining research support than the other two groups (p = 0.001). While resident doctors reported being the least competent, specialist doctors also felt less competent than academicians (p = 0.001).

In our study, single doctors stated that they had less competence (p = 0.005) and research support (p = 0.007) than married doctors. There was no difference between them in terms of motivation (p = 0.389) and time constraints (p = 0.507). At the time of our study, 76.4% of single respondents were between the ages of 25 and 34 years, while 37.4% of married people were between ages 25 and 34.

Participants who work in a private hospital stated that they were less motivated than those working in a university hospital (p = 0.004). In addition, physicians working in state hospitals stated that they receive less research support than doctors working in universities and private hospitals (p = 0.002).

Considering geographic regions, there were no differences among the four zones in terms of time constraint, research support, and competence. However, physicians in the fourth zone stated that they were less motivated than the others (p = 0.010).

There was no significant difference in any parameter for those who received their residency training in university versus state hospitals. However, considering the geographic regions, doctors who received residency training in the first zone appeared to be significantly more motivated than others (p = 0.026). Also, those who received residency training in the second zone considered themselves slightly more competent than those in other regions (p = 0.054).

Table 4 shows the correlation analysis of the factors on the research barriers scale. As shown, those with high competence have higher motivation. Likewise, those with high competence can access research support more easily. It was found that those who can easily access research support have less time constraints.

**Table 4:** Research barriers scale factors correlation analysis.

	Motivation	Time Constraints	Research Support	Competence
**Motivation**	1			
**Time Constraints**	-,085	1		
**Research Support**	,090	-,180(**)	1	
**Competence**	,301(**)	-,065	,162(*)	1

Pearson Correlation Analyses. * Correlation is significant at the 0.05 level (2-tailed) ** Correlation is significant at the 0.01 level (2-tailed)

## 4. Discussion

Our study investigated the challenges of conducting SR using an online questionnaire distributed to ophthalmologists in Turkey. The results showed that ophthalmologists are generally motivated to do SR, but they encounter certain obstacles. Most participants stated that they need the more time, institutional research support, and training related to the research process and writing articles. Also, the majority complained about the complexities of research and publication procedures.

In our study, we found greater interest of the participants in cataract and refraction surgery followed by medical retina. Akmaz et al. [15] analyzed the articles published in Scientific Citation Index (SCI) ophthalmology journals from Turkey between January 2014 and September 2019. They found that the number of publications in the medical retina field was highest, at 32.4%. The rate of publications in the field of cataract and refractive surgery was only 4.3%. This shows that the interest in cataract and refractive surgery is not reflected in the number of publications. In our study, participants stated that they are interested in oculoplastic surgery (ranked in third place). In the study by Akmaz et al. [15], the rate of publications on oculoplastic surgery was only 2.9%. Again, the interest in oculoplastic surgery is not reflected in the number of publications. Widespread application of cataract surgery and the lack of motivation to publish among oculoplastic surgeons are notable in these results.

Studies on research productivity have focused on sex disparities in recent years. Women’s personal responsibilities, such as raising children, and systematic academic barriers put them at a disadvantage in scientific activities that require intense commitment and continuity [16]. In the field of ophthalmology, studies have shown that women still face discriminatory attitudes in terms of academic prestige and career advancement, despite the relative balance in the number of men and women experts in recent years [10, 17–19]. In our study, women were shown to be more motivated than men to do SR. Also, women reported statistically significantly more obstacles associated with their personal responsibilities on the seventh question. One reason for this difference may be the responsibility to take care of other people at home. Jolly et al. [20] revealed the disadvantage of women in their wide-ranging study that investigated the difference by gender in time spent by successful young physician-researchers on parenting and domestic responsibilities. However, in our study, this issue could not be verified because we did not question the presence of a person in need of care, such as a child or an elderly person, at home. In addition, interestingly, female doctors stated in the 21st question that the difficulty of the publishing process of scientific articles inhibited their motivation. Kramer et al. [10] analyzed 87,640 articles published between January 2008 and August 2018 in the ophthalmic research area. They found lower rates of authorship for women as the first author and co-author. One reason for women’s underrepresentation may be the lower acceptance rate by journals of women’s manuscripts than of men’s. Hence, this may explain more clearly why the publishing process discourages women. There are journal-based studies on acceptance rates by gender [21, 22]. However, we could not find a current study showing the acceptance rates of women’s manuscripts by ophthalmology journals. This issue needs further evidence.

The negative effects of age on scientific productivity is well established in the literature [23]. Our study reflected that the motivation to conduct SR decreases with age. It has been previously shown that the desire for promotion increases academic performance [24]. Therefore, a decline in academic career-related promotion expectations at later ages explains this decrease in motivation. Similarly, motivation is likely to decrease due to weariness that accumulates over time in the face of difficult challenges encountered.

According to our study, academicians have an advantage over specialist doctors in acquiring research support. In addition, depending on the status of the institution, it was observed that those working in state hospitals have more difficulty finding research support than those working in other institutions. These two data support each other. The study by Akmaz et al. [15] found that universities and training-research hospitals, which are generally where academicians work, were far ahead of state hospitals, where specialist doctors work, in terms of research productivity. The data in our study elucidate this difference between academicians and specialists, or between university and state hospitals. We found in the correlation analysis that participants who could find research support were more competent and had fewer time constraints. We think the answers given by academicians play an important role in this; they reported having fewer time constraints than other doctors that conflict with their participation in SR.

Physicians in the 4th region did not show a significant difference in time constraints, research support, and competence from those in other zones. However, they stated that they were less motivated to do SR. SR requires scientific collaboration, and the impact of geography on scientific collaboration has long been of interest to researchers [25, 26]. Accordingly, greater distances are a factor that reduces scientific collaboration. The distance between research units in zone 4 is higher than in other geographic regions in Turkey. We think that this motivation problem in zone 4 is primarily due to the difficulty in arranging research collaboration.

Physicians who received their residency training in the first zone were more motivated, while those who received it in the second zone felt more competent. Demirtas et al. [12] investigated the academic productivity of orthopedic specialists according to geographic regions where they received their residency training. They found that those who received training in the Central Anatolia region (second zone) were more productive, with a significant difference. When we evaluate these findings with the data in our study, we can say that residency training in the Central Anatolia region encourages more scientific activity and increases competence in these two surgical branches. Another striking point in our study was that physicians generally continued their careers in the regions where they received residency training. Zone 1, the Marmara region, is where research centers are most concentrated in Turkey, and it is easier to establish research collaboration in this region compared to others. We think that physicians trained in zone 1 are more motivated due to this advantage. Most of these physicians work in the first zone.

This study has some limitatitons. We achieved a relatively low number of participants, although the profile of participants powerfully reflects the average in Turkey. The reason we reached a smaller number of participants may be reluctance on the part of the participants due to the increasing number of online surveys. A second limitation is failure to ask physicians the number of their dependents at home.

## 5. Conclusion

This study evaluates the barriers to SR for ophthalmologists in Turkey from many angles, especially gender, in order to increase scientific productivity. Accordingly, while ophthalmologists stated that they are highly motivated to conduct SR, they mentioned a series of obstacles. These obstacles can mainly be summarized as not having enough time and not getting research support. In addition, the need to increase competence in SR is a major finding of this study. Although in our study, women are more motivated to do SR than men, traditional responsibility burdens continue to challenge them. However, there was no statistically significant difference between women and men in terms of time availability, research support, and competence. As a result, managers responsible for improving academic productivity should take into account the barriers identified herein and seek ways to alleviate them. In this way, SR productivity in the field of ophthalmology will be the ultimate beneficiary.

## Supporting information

S1 Table The Turkish version of the survey. (DOCX)

S2 Table The English version of the survey. (DOCX)
