# Supplementary material for: Evaluating scientific research barriers by gender and other characteristics from the perspective of ophthalmologists in Turkey: A multicenter survey study
Source: PLoS One. 2023 Jan 25;18(1):e0273181. doi: 10.1371/journal.pone.0273181 (PMC9876363; doi:10.1371/journal.pone.0273181)
Supplement: S1 Table — (DOCX) [file pone.0273181.s001.docx]

**Sayın Katılımcı**

Bu çalışmada göz hekimlerinin akademik çalışmalarla ilgili yaşadığı zorlukları tespit etmek amaçlanmıştır. Bu anket formu sonucunda elde edilecek veriler tamamen bilimsel çalışma amaçlı kullanılacaktır. Anketimize ayırdığınız kıymetli zamanınız, saygıdeğer emeğiniz, samimi ve içten cevaplarınız için teşekkür ederiz.

**Dr. Öğr. Üyesi Burak Erdem**

Araştırma Yürütücüsü

| **Statünüz**  🞏 Asistan Hekim 🞏 Uzman Hekim 🞏 Dr. Öğretim Üyesi 🞏 Doçent 🞏Profesör |
| --- |
| **Cinsiyetiniz**  🞏Kadın 🞏 Erkek |
| **Yaşınız**  🞏 25-34 🞏 35-44 🞏 45 ve yukarısı |
| **Medeni durumunuz**  🞏 Bekar 🞏 Evli |
| **Göz doktoru olarak çalışma yılı (Asistanlık süresi dahil)**  🞏 1-5 yıl  🞏 6-10 yıl  🞏 11–15 yıl  🞏 16 yıl ve üzeri |
| **Çalıştığınız Kurum**  🞏 Sağlık Bakanlığı Hastanesi  🞏 Devlet Üniversite Hastanesi  🞏 Özel Hastane |
| **Çalıştığınız Kurumun Coğrafik Bölgesi**  🞏 Marmara Bölgesi  🞏 İç Anadolu Bölgesi  🞏 Ege Bölgesi  🞏 Akdeniz Bölgesi  🞏 Karadeniz Bölgesi  🞏 Doğu Anadolu Bölgesi  🞏 Güneydoğu Anadolu Bölgesi |
| **Asistanlık Eğitiminizi Tamamladığınız Kurum**  🞏 Sağlık Bakanlığı Hastanesi  🞏 Üniversite Hastanesi |
| **Asistanlık Eğitiminizi Tamamladığınız Kurumun Coğrafik Bölgesi**  🞏 Marmara Bölgesi  🞏 İç Anadolu Bölgesi  🞏 Ege Bölgesi  🞏 Akdeniz Bölgesi  🞏 Karadeniz Bölgesi  🞏 Doğu Anadolu Bölgesi  🞏 Güneydoğu Anadolu Bölgesi |

|  |  | **Katılma Düzeyi** | | | | |
| --- | --- | --- | --- | --- | --- | --- |
| **No** | **Göz Hekimlerinin Akademik Üretkenlik Ölçeği** | **Hiç Katılmıyorum** | **Katılmıyorum** | **Kararsızım** | **Katılıyorum** | **Tamamen Katılıyorum** |
|  | Bilimsel çalışma yapmaya istekliyim |  |  |  |  |  |
|  | Bilimsel çalışma yapmak için ciddi çaba harcıyorum |  |  |  |  |  |
|  | Bilimsel yayın yapmayı mesleki zorunluluklar çerçevesinde değerlendiriyorum |  |  |  |  |  |
|  | Bilimsel çalışma ve yayınlar mesleki kariyerime önemli bir katkı sağlıyor |  |  |  |  |  |
|  | Hasta yoğunluğu bilimsel çalışma yapmamı önemli ölçüde sınırlıyor |  |  |  |  |  |
|  | Hastanede hasta muayenesi/tedavisi dışındaki diğer prosedürler (veri kaydı girmek, dosya doldurmak vs.) bilimsel çalışma yapmak için zaman ayırmamı engelliyor |  |  |  |  |  |
|  | Kişisel işlerim ve sorumluluklarım bilimsel çalışmaya zaman ayırmamı zorlaştırıyor |  |  |  |  |  |
|  | Bilimsel çalışma yapmak kurum yönetimi tarafından desteklenmiyor |  |  |  |  |  |
|  | Yöneticilerim tarafından çalışma saatleri içinde bilimsel çalışmalar için ayrılmış özel zamanım bulunmaktadır |  |  |  |  |  |
|  | Asistanlık eğitimim boyunca bilimsel çalışma yapmaya yönlendirildim/yönlendiriliyorum |  |  |  |  |  |
|  | Çalıştığım kurumun teknik altyapısı bilimsel çalışma yapmak için yeterlidir |  |  |  |  |  |
|  | Bilimsel yayınların teşvik edilmesine yönelik düzenlemeler ve maddi destekler yeterlidir |  |  |  |  |  |
|  | Bilimsel çalışma yapmak için yeterli maddi desteklere sahibim |  |  |  |  |  |
|  | Bilimsel toplantılara katılmak için yeterli ekonomik destek bulabiliyorum |  |  |  |  |  |
|  | Bilimsel çalışma yapmak için yeterli İngilizce bilgisine sahibim |  |  |  |  |  |
|  | Bilimsel çalışma yapmak için yeterli istatistik bilgisine sahibim |  |  |  |  |  |
|  | “Bilimsel çalışma nasıl yapılır?” konusunda eğitime ihtiyacım yoktur |  |  |  |  |  |
|  | “Makale nasıl yazılır?” konusunda eğitime ihtiyacım yoktur |  |  |  |  |  |
|  | Sosyal medyada geçirdiğim zaman bilimsel çalışmaya ayırdığım zamanı azaltmaz |  |  |  |  |  |
|  | Bilimsel çalışma yapmak için gerekli prosedürler bezdirici olabiliyor |  |  |  |  |  |
|  | Bilimsel çalışmaları yayınlatma sürecinin yorucu ve yıpratıcı olması motivasyonumu azaltıyor |  |  |  |  |  |

Açık Uçlu Sorular;

1. Sizi akademik üretime motive eden üç önemli nedeni yazınız.

………….

1. Akademik çalışma yapmanızı zorlaştıran üç önemli nedeni yazınız.

…………..

Teşekkürler
