# Supplementary material for: Evaluating scientific research barriers by gender and other characteristics from the perspective of ophthalmologists in Turkey: A multicenter survey study
Source: PLoS One. 2023 Jan 25;18(1):e0273181. doi: 10.1371/journal.pone.0273181 (PMC9876363; doi:10.1371/journal.pone.0273181)
Supplement: S2 Table — (DOCX) [file pone.0273181.s002.docx]

**Dear Participant**

This study aims to identify the difficulties related to the academic studies of ophthalmologists. The data obtained as a result of this survey form will be used for scientific study purposes only. Thank you for your valuable time, your esteemed effort, and your sincere and sincere answers to our survey.

**Asst. Prof. Dr. Burak Erdem**

Research Coordinator

| **Title**  🞏 Ophthalmology Residency 🞏 Ophthalmology Specialist 🞏 Asst. Prof. 🞏 Assoc. Prof. 🞏Professor |
| --- |
| **Gender**  🞏Female 🞏 Male |
| **Age**  🞏 25-34 🞏 35-44 🞏 45 and over |
| **Marital Status**  🞏 Single 🞏 Married |
| **Working year as an ophthalmologist**  🞏 1-5 years  🞏 6-10 years  🞏 11–15 years  🞏 16 years and up |
| **Institution**  🞏 State Hospital  🞏 University Hospital  🞏 Private Hospital |
| **The geographic region of your institution**  🞏 Marmara Region  🞏 Central Anatolia Region  🞏 Aegean Region  🞏 Mediterranean Region  🞏 Black Sea Region  🞏 Eastern Anatolia Region  🞏 Southeastern Anatolia Region |
| **The status of the institution where you completed the residency training**  🞏 State Hospital  🞏 University Hospital |
| **The geographical region of the institution where you completed the residency training**  🞏 Marmara Region  🞏 Central Anatolia Region  🞏 Aegean Region  🞏 Mediterranean Region  🞏 Black Sea Region  🞏 Eastern Anatolia Region  🞏 Southeastern Anatolia Region |

|  |  | **Confirmation Level** | | | | |
| --- | --- | --- | --- | --- | --- | --- |
| **No** | **The Research Barriers Scale of Ophthalmologists** | **Fully Disagree** | **Disagree** | **Neither disagree nor agree** | **Agree** | **Fully Agree** |
|  | I am enthusiastic to do scientific research |  |  |  |  |  |
|  | I make serious efforts to do scientific research |  |  |  |  |  |
|  | Scientific research is a professional obligation |  |  |  |  |  |
|  | Scientific research and publications make an important contribution to my academic career |  |  |  |  |  |
|  | The excess of patients in the clinic prevents my scientific researches |  |  |  |  |  |
|  | Procedures such as electronic health records reduce my time spent on scientific research |  |  |  |  |  |
|  | My personal works and responsibilities reduce my time devoted to scientific research |  |  |  |  |  |
|  | Scientific research is sufficiently supported by the institution's management |  |  |  |  |  |
|  | I have a special time reserved for scientific research during working hours |  |  |  |  |  |
|  | During the residency training, I was encouraged to do scientific research |  |  |  |  |  |
|  | The technical infrastructure of my institution is suitable for scientific research |  |  |  |  |  |
|  | Regulations and financial support to encourage scientific publications are sufficient |  |  |  |  |  |
|  | I have sufficient financial support to do scientific research |  |  |  |  |  |
|  | I have sufficient financial support to attend academic meetings |  |  |  |  |  |
|  | I have sufficient knowledge of English to do scientific research |  |  |  |  |  |
|  | I have sufficient knowledge of statistics to do scientific research |  |  |  |  |  |
|  | I don't need training on "How is scientific research done?" |  |  |  |  |  |
|  | I do not need training on "How is the article written?" |  |  |  |  |  |
|  | Using social media does not take away from the time I spend doing scientific research |  |  |  |  |  |
|  | The procedures required to do scientific work can be tedious |  |  |  |  |  |
|  | The tiring process of publishing scientific studies reduces my motivation |  |  |  |  |  |

Open-ended Questions;

1. Write down three important reasons that motivate you to academic production.

………….

1. Write down three important reasons why you find it difficult to do academic studies.

…………..

Thanks
